# Airway management in simulated restricted access to a patient - can manikin-based studies provide relevant data?

**DOI:** 10.1186/1757-7241-19-36

**Published:** 2011-06-13

**Authors:** Anders R Nakstad, Mårten Sandberg

**Affiliations:** 1 Air Ambulance Department, Oslo University Hospital, Sykehusveien 19, N-1474 Nordbyhagen, Norway; 2 University of Oslo, Oslo, Norway

## Abstract

**Background:**

Alternatives to endotracheal intubation (ETI) are required when access to the cranial end of the patient is restricted. In this study, the success rate and time duration of standard intubation techniques were compared with two different supraglottic devices. Two different manikins were used for the study, and the training effect was studied when the same manikin was repeatedly used.

**Methods:**

Twenty anaesthesiologists from the Air Ambulance Department used iGEL^™^, laryngeal tube LTSII^™ ^and Macintosh laryngoscopes in two scenarios with either unrestricted (scenario A) or restricted (scenario B) access to the cranial end of the manikin. Different manikins were used for ETI and placement of the supraglottic devices. The technique selected by the physicians, the success rates and the times to completion were the primary outcomes measured. A secondary outcome of the study was an evaluation of the learning effect of using the same manikin or device several times.

**Results:**

In scenario A, all anaesthesiologists secured an airway using each device within the maximum time limit of 60 seconds. In scenario B, all physicians secured the airway on the first attempt with the supraglottic devices and 16 (80%) successfully performed an ETI with either the Macintosh laryngoscope (n = 13, 65%) or with digital technique (n = 3, 15%). It took significantly longer to perform ETI (mean time 28.0 sec +/- 13.0) than to secure an airway with the supraglottic devices (iGel™: mean 12.3 sec +/- 3.6, LTSII™: mean 10.6 sec +/- 3.2). When comparing the mean time required for the two scenarios for each supraglottic device, there was a reduction in time for scenario B (significant for LTSII^™^: 12.1 versus 10.6 seconds, p = 0.014). This may be due to a training effect using same manikin and device several times.

**Conclusions:**

The amount of time used to secure an airway with supraglottic devices was low for both scenarios, while classic ETI was time consuming and had a low success rate in the simulated restricted access condition. This study also demonstrates that there is a substantial training effect when simulating airway management with airway manikins. This effect must be considered when performing future studies.

## Background

Fast and safe airway management in the field is critical but sometimes challenging due to patient and environmental factors. Airway management in entrapped patients or patients located in a confined space can be especially demanding. Inadequate lighting and impaired access to the patient add to the complexity to such situations and increase the risk of adverse events [1]. Attempts at endotracheal intubation (ETI) under suboptimal conditions should be avoided, and safer alternatives should be used whenever possible [2,3]. Reports from use of supraglottic devices in simulated restricted access and in cases of resuscitation or unanticipated difficult airway are promising [4-6]. Some investigators, however, have reported the successful use of inverse intubation techniques in trauma patients. In a simulated scenario of inverse intubation during helicopter-flight similar time consumption in the interval of 21-24 seconds was reported for classical ETI and inverse technique [7].

Supraglottic devices represent an alternative to ETI. In our prehospital service, a laryngeal tube with a suction canal (LTSII^™^) is the most frequently used supraglottic device until now; it is used both as a primary device and as a backup device if ETI fails [8]. A multitude of devices are commercially available, and the superiority of one device has not been established. The widespread use of supraglottic devices by emergency medical services is due to the relatively high placement success rates [9]. Importantly there seems to be a difference in what is reported as success rates in manikin studies and in real patients [10]. In a few cases, supraglottic devices have been reported to have been used prior to hospital arrival to secure an airway in trauma patients with limited airway access [11].

The aim of this study was to compare the use of iGEL^™ ^and LTSII^™ ^with ETI in manikins in settings designed to mimic airway management in entrapped patients.

## Methods

### Study design and participants

The twenty study participants were specialists in anaesthesiology employed by the Air Ambulance Department at the Oslo University Hospital and they participated voluntarily.

None of the participants had extensive experience with the iGel^™ ^(Intersurgical Ltd., Wokingham Berkshire, UK) device in a clinical setting prior to this study. Only five of the participants had used it clinically within the previous two years. All participants were familiar with the LTSII^™ ^(VBM Medizintechnik GmbH, Sulz a.N., Germany) as a backup device, but only two had used it clinically within the previous two years.

Based on preliminary testing, the Airsim Standard^™ ^(Truecorp Ltd., Belfast, UK) manikin head was selected for intubation procedures and the Airway Management Trainer^™ ^(Ambu Ltd., St. Ives, UK) manikin head was selected for use with supraglottic devices. The main criteria for choosing the two manikins was that we were able to demonstrate little variability in insertion times with identical techniques performed by the same person. Older manikins demonstrated high variability in insertion times and thus were regarded as unfit for this study.

To evaluate the training effect of using standardised manikins, the order of device placement was not randomised. The iGel^™ ^was placed first, followed by the LTSII^™ ^device and then ETI was performed. The sequence was first made in scenario A (optimal conditions) and then repeated in scenario B (restricted access).

### Study protocol

**Figure 1:**
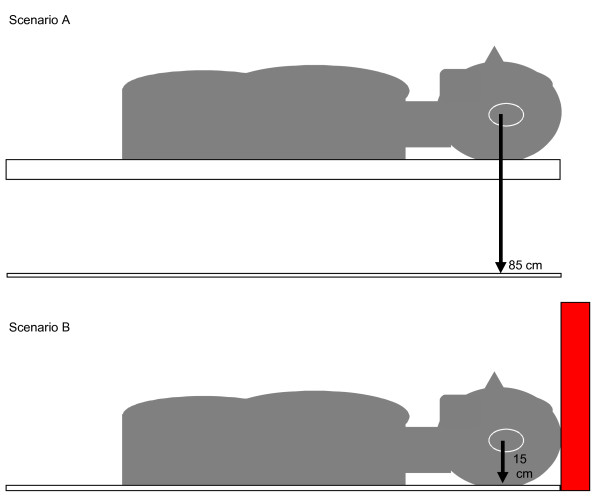
**Arrangement of manikins for simulated optimal and restricted access**. Legend (figure 1): In scenario A the manikin heads were placed on a table 85 cm above the ground with unrestricted access from the head end. In scenario B the manikin heads were placed on the ground with the cranial end in contact with a wall making access from the head end impossible.

In scenario A, the manikins were placed on an 85-cm high table, which corresponded to the working height of a patient on an ambulance stretcher (Figure 1). This scenario was intended to represent the typical setting for controlled prehospital airway management. In scenario B, the manikins were placed on the ground abutting a wall, and access to the manikin head and airway was from the caudal end only. This setting was arranged to mimic restricted access conditions encountered when patient airway management must be performed prior to evacuation of the patient from a wreck or confined space.

The number of attempts, the time spent to secure an airway and the technique selected were the primary outcome variables. The start time was defined as when the anaesthesiologist was asked to begin while standing one meter away from the manikins with the equipment in hand, and the end of the procedure was defined as when the physician verbally stated that the airway was secured. For LTSII and endotracheal tubes this time interval included inflation of the cuff. The placement of the device was then visually inspected and proper placement verified by connecting a self-inflatable bag controlling that the artificial lungs were adequately inflated with no air leakage from the manikin.

An unsuccessful procedure was defined as an attempt that did not result in a secured airway within 60 seconds from starting. Use of digital technique in ETI was accepted if it was chosen by the participant.

Scenario A was performed prior to scenario B for all participants.

### Data analysis

Data were analyzed using the spreadsheet Excel (Microsoft, Redmond, WA, USA), and the statistical package EPI-info version 3.5.1 (Centre for Disease Control (CDC), Atlanta, GA, USA). The chi square test and Fisher's exact test were used for comparing frequencies. Wilcoxon's paired-t test was employed for other nonparametric data.

## Results

### Scenario A (optimal access)

In scenario A, all anaesthesiologists secured an airway using each device well within the maximum time limit of 60 seconds. There were no significant differences in the time to completion using the iGel^™^, LTSII^™ ^or ETI devices (Table 1).

**Table 1:** Mean time used to insert supraglottic devices and endotracheal tube in simulated optimal and restricted access

Device	Manikin	Scenario	Number	Successful	Mean time (seconds)	SD
iGel ™	Ambu ™	A (optimal)	20	All	9.9	4.5
iGel ™	Ambu ™	B (restricted)	20	All	12.3	3.6
LTSII ™	Ambu ™	A (optimal)	20	All	12.8	2.9
LTSII ™	Ambu ™	B (restricted)	20	All	10.6	3.2
						
Macintosh #3	TrueCorp ™	A (optimal)	20	Yes	12.1	3.3
			0	No		
Macintosh #3	TrueCorp ™	B (restricted)	16	Yes	28.0	13.0
			4	No		
						
**P-values for comparing same device in scenario A versus B**						
Mean time with iGel in scenario A vs scenario B				p = 0.09	NS	
Mean time with LTSII in scenario A vs scenario B				p = 0.01	S	
Mean time with Macintosh laryngoscope (blade #3) in scenario A vs Scenario B				p < 0.01	S	
						
**P-value for comparing devices with each other in scenario A**						
Mean time with iGel vs LTSII				p = 0.69	NS	
Mean time with Macintosh #3 vs iGel				p = 0.88	NS	
Mean time with Macintosh #3 vs LTSII				p = 0.19	NS	
						
**P-values for comparing differen devices with each other in scenario B**						
Mean time with iGel vs LTSII				p = 0.50	NS	
Mean time with Macintosh #3 vs iGel				p < 0.001	S	
Mean time with Macintosh #3 vs LTSII				p < 0.001	S	
						
NS = Non-significant, S = significant						

Legend (table 1): The success rates and mean time (seconds) used to insert the supraglottic device and endotracheal tube in simulated optimal (scenario A) and restricted (scenario B) access conditions. Relevant P-values are listed. Specific comment for Macintosh #3 in scenario B: three HEMS physician chose to use digital technique when inserting the endotracheal tube. In 13 cases classic laryngoscopy technique succeeded. In the remaining four cases of attempted direct laryngoscopy no endotracheal tube was placed within the time limit of 60 seconds.

### Scenario B (restricted access)

In scenario B, all physicians secured the airway on the first attempt with the supraglottic devices but only 16 (80%) successfully performed an ETI with either the Macintosh laryngoscope (n = 13, 65%) or with digital technique (n = 3, 15%). It took significantly longer to perform ETI than to secure an airway with the supraglottic devices in this scenario (p < 0.001). No participants reported that they were comfortable with the ETI procedure under the limited access conditions, and only three stated that they were certain the endotracheal tube was correctly placed in the trachea of the manikin head. Two of these three physicians used the digital technique.

For scenario B, all physicians secured an airway on their first attempt when using the supraglottic devices. When comparing the mean times for device placement, we observed a reduction in time for scenario B compared to scenario A of 2.2 seconds (p = 0.01) for the LTSII^™ ^and an increase in time for scenario B compared to scenario A of 2.4 seconds (p = 0.19) for the iGel^™^.

### Discussion

#### Main findings

Our results show that airway management with iGel^™^, LTSII^™ ^and ETI in scenarios with optimal access to the simulated patient (scenario A) is fast and has high success rates with all devices when performed by experienced anaesthesiologists. The difference in time spent between the devices is probably of no clinical significance. Thus, with optimal access to the patient, ETI is the method of choice, because it results in a cuffed tube in the trachea.

In a scenario of restricted access to the manikin head (scenario B), however, our study indicates that ETI is potentially unsafe with four of 20 attempts not resulting in a secured airway. ETI was also a more time-consuming technique under these conditions, although an increase of 16 seconds may not be clinically significant. Based on the results from scenario B, one could argue that supraglottic devices are superior to ETI when the access to the patient's airway is restricted.

#### Relevance of topic

Under ideal conditions, experienced physicians can perform ETI prehospitally with similar success rates as when performed in the hospital [12-14]. Usually, the patient can be evacuated onto an ambulance stretcher with an adjustable height to improve the environmental conditions prior to definitive airway management. However, entrapped patients and patients located in confined spaces may occasionally be in such respiratory distress that a secure airway and mechanical ventilation prior to extrication or transport are required. In a multi-center study from German HEMS, by Helm and co-workers, limited access to the patient was found in 20% of patients upon arrival and in almost 10% of patients at the time of the first intubation attempt [1]. This makes it relevant to study if supraglottic devices provide a safer way to secure the airway in cases of restricted access.

#### Use of manikin studies

Recent years have provided numerous studies on equipment and techniques evaluated by use in manikins - a trend that has been strongly criticised [15]. We believe manikin studies can be useful for evaluating techniques where tissue quality is of little importance - like in the evaluation of video laryngoscopes and fibre scopes [2,16,17]. In addition, in studies like the present study of airway management in patients where the access is restricted, manikins are needed for ethical reasons. However, as mentioned below, a manikin-based study must be well-designed to become an acceptable surrogate for real patients.

### Limitations of this study

One previous study, and our early testing prior to this study, indicated that there may be a training effect when the same airway simulator is used for a limited number of airway manoeuvres [9]. To evaluate this possible effect we decided not to randomize the sequence of the techniques performed in the two different scenarios. In addition, scenario B was constructed so that a significant increase in time spending could be anticipated if there was no training effect. The finding of a small significant reduction in the mean time spent on securing the airway of the manikin with LTSII^™ ^between scenario A and B, despite the much higher degree of difficulty in scenario B, support our assumption of a substantial training effect. It is possible that the participants remembered the anatomy and tissue-quality of the manikins in scenario A such that repeat testing in scenario B resulted in faster completion times. It may also, however, be that the increased familiarity with the LTSII^™ ^is the main reason. Some studies have evaluated the role of different airway trainers when teaching how to place supraglottic devices [18,19]. One recent study compared the use of fresh frozen cadavers with selected airway simulators to evaluate which simulator mimicked the quality of a real intubation [20]. None of these studies, however, addressed the implications of a fixed anatomical condition.

The need to employ two different manikins is a significant limitation of this study. However, we believe that the limitations of the study would have been more significant if only one manikin had been used, because we found no manikin suitable for both types of simulated airway intervention. The arrangements of the manikins were made as similar as possible.

## Conclusions

Airway management in cases of restricted patient access is not emphasised in current airway management guidelines [21-23].

Based on use of a manikin head, this study demonstrates that ETI is potentially unsafe in a scenario of restricted access to a patient. Supraglottic devices seem superior. No clinically important difference was found between the two devices studied.

Our study indicates that a substantial training effect exists after just two manoeuvres with an airway simulator and two different airway devices. This effect is likely due to the fixed anatomy and material of the manikins. It must be considered when evaluating different airway management techniques and airway devices in future studies.

## Competing interests

No author has any conflict of interest with regard to the material being discussed in this manuscript.

## Authors' contributions

ARN and MS participated in the design and writing of the manuscript. ARN performed the data sampling and statistical analysis. Both authors read and approved the final manuscript.
